# Management and Outcomes of Acute Respiratory Distress Syndrome Caused by Blastomycosis: A Retrospective Case Series

**DOI:** 10.1097/MD.0000000000003538

**Published:** 2016-05-06

**Authors:** Ilan S. Schwartz, John M. Embil, Atul Sharma, Stephen Goulet, R. Bruce Light

**Affiliations:** From the Department of Medical Microbiology (ISS, JME, RBL); Department of Medicine (ISS, JME, RBL), Section of Infectious Diseases, College of Medicine, University of Manitoba, Winnipeg, Manitoba, Canada; Department of Epidemiology and Social Medicine (ISS), Faculty of Health Sciences, University of Antwerp, Antwerp, Belgium; Biostatistical Consulting Unit (AS), George and Fay Yee Center for Healthcare Innovation, University of Manitoba; Department of Pediatrics and Child Health (AS), Section of Nephrology; Department of Medicine (SG), Section of General Internal Medicine; and Department of Medicine (RBL), Section of Critical Care Medicine, College of Medicine, University of Manitoba, Winnipeg, Manitoba, Canada.

## Abstract

Acute respiratory distress syndrome (ARDS) is an uncommon, highly fatal, and poorly understood manifestation of blastomycosis. Optimal management remains unknown, including the roles of adjunctive corticosteroids and extracorporeal membrane oxygenation (ECMO).

We conducted a retrospective chart review of patients with ARDS caused by blastomycosis, managed in intensive care units in Manitoba, Canada, from 1992 to 2014. ARDS was defined using the Berlin definition. Corticosteroid therapy was defined as ≥150 mg cortisol equivalent in 24 hours. Logistic regression was used to identify determinants of a fatal outcome, and bootstrap resampling was used to assess sample size requirements.

Forty-three patients with ARDS caused by blastomycosis were identified. ARDS was mild, moderate, and severe in 2 (5%), 12 (28%), and 29 (67%) patients, respectively. Management included amphotericin B (n = 42, 98%), vasopressors (n = 36, 84%), corticosteroids (n = 22, 51%), renal replacement (n = 13, 30%), and ECMO (n = 4, 11%). Seventeen patients (40%) died. All patients treated with ECMO survived (*P* = 0.14). Corticosteroids were not associated with survival benefit in univariate (*P* = 0.43) or multivariate analyses (odds ratio 0.52, 95% confidence interval 0.11–2.34). Bootstrap studies indicated that almost 500 patients would be needed to confirm a significant reduction in mortality from corticosteroids (type I error = 0.05, power = 80%).

Blastomycosis is an uncommon, albeit important, cause of ARDS in this geographic area. Given the rarity of disease and the large cohort needed to demonstrate mortality benefit, the role of adjunctive therapies, including corticosteroids and ECMO, may remain unconfirmed, and clinical judgment should guide management decisions.

## Abstract 2

Supplemental Digital Content is available in the text

## INTRODUCTION

Blastomycosis is an uncommon granulomatous disease caused by infection with thermally dimorphic fungi of the genus *Blastomyces*.^1^ The spectrum of lung disease in blastomycosis ranges from subclinical to severe pneumonia, including the acute respiratory disease syndrome (ARDS), which may complicate up to 15% of cases, and which carries a mortality rate in excess of 50%.^2–5^ Although antifungals—particularly amphotericin B formulations—form the mainstay of management, the roles of adjuvant therapies, including corticosteroids and venovenous extracorporeal membrane oxygenation (ECMO), are uncertain.^1,6,7^ Although the clinical guidelines of the Infectious Diseases Society of America for the management of patients with blastomycosis do not recommend for or against adjunctive corticosteroids,^6^ those of the American Thoracic Society suggest considering adjunctive corticosteroids for patients with blastomycosis pneumonia and severe gas exchange abnormalities.^7^ However, only 2 cases^8^ are cited as evidence for this recommendation. Neither guidelines discuss ECMO in the management of ARDS caused by blastomycosis.^6,7^ Although several case series of ARDS caused by blastomycosis have been reported,^2–5^ the numbers of patients have been limited, and the role of adjunctive therapies for this disease has not been examined.

In this study, we describe the epidemiology, clinical characteristics, management, and outcomes of patients with ARDS caused by blastomycosis, who received care in Manitoba, Canada intensive care units (ICUs), which provide referral services to an area of hyperendemicity for blastomycosis.^9^ We hypothesized that corticosteroids would be associated with survival advantage.

## METHODS

This study was a retrospective chart review of patients with ARDS caused by blastomycosis, who received care in Manitoba ICUs over a 23-year period. The ICU sites involved in this study included 2 tertiary and 4 community hospitals in Winnipeg, and 1 community hospital in Brandon, Manitoba. Ethics approval was granted by the Human Research Ethics Board of the University of Manitoba, and by the institutional review boards of each hospital.

The catchment areas for these ICUs include the province of Manitoba, and parts of north-western Ontario, Canada. A prior study established the incidence of blastomycosis in these areas as 0.62 and 7.11 cases per 100,000 population, respectively.^9^

Patients with ARDS caused by blastomycosis occurring between January 1992 and September 2014 were identified through retrospective laboratory and clinical surveillance systems: we reviewed *Blastomyces* spp. isolates for patients managed in an ICU, and an administrative database of ICU patients for those with proven diagnoses of blastomycosis. All diagnoses were proven with cytopathological examination and/or culture of clinical specimens. Serological or antigen tests were not utilized for diagnosis. Patients were excluded if they had concurrent, acute illnesses that precluded clear attribution of ARDS to blastomycosis.

Acute respiratory distress syndrome was defined and graded according to the Berlin criteria,^10^ summarized in Table 1. Indices of hypoxemia (arterial oxygen pressure to fraction of inhaled oxygen ratio) and ventilation (positive end expiratory pressure) within 48 hours of ICU admission were recorded. When applicable, initial pulmonary artery catheter measurements were noted. Acute Physiology and Chronic Health Evaluation II (APACHE II) scores^11^ were calculated within 24 hours of ICU admission. Shock was defined as persistent hypotension, despite adequate fluid resuscitation, requiring vasopressors to maintain end-organ perfusion. Corticosteroid therapy was defined as ≥150 mg of cortisol equivalent (using published conversion tables^12^) within a 24-hour period, before clinical stabilization. The term Aboriginal was used to refer to Canadians who identified themselves to be of First Nations, Métis, or Inuit ancestry.^13^ Patients were considered to have survived if discharged from the ICU in stable condition; this included transfer to the ward or repatriation to a community hospital for convalescence.

**TABLE 1:**
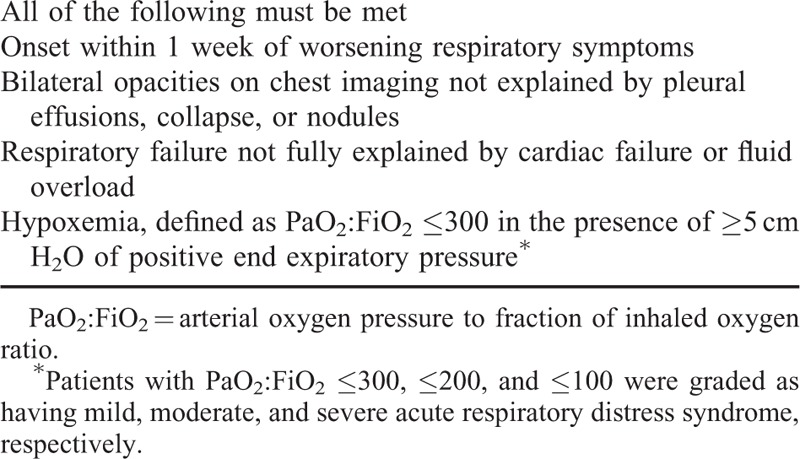
Berlin Criteria for Acute Respiratory Distress Syndrome

Statistical analysis included univariate analyses without adjustments for multiple comparisons using Fisher exact and Mann–Whitney *U* tests for categorical and nonparametric, continuous variables, respectively. *P* values ≤0.05 were considered significant. These analyses were performed using SPSS version 22 (IBM Corp., Armonk, NY).

To assess the impact of corticosteroid therapy on mortality, a multivariate logistic regression was also performed, with adjustment for variables that emerged as significant in our univariate analysis (critical illness severity and underlying cardiopulmonary disease). Routine diagnostics were performed for all regression models using standard methods.^14^

To determine the sample size needed for this retrospective study to demonstrate significant effect of corticosteroids on mortality, a nonparametric bootstrap resampling procedure was performed, assuming that our outcome data are representative in terms of comorbidity (cardiopulmonary disease), disease severity (APACHE II), and treatment effect (corticosteroids). Briefly, for sample sizes ranging from 100 to 1000 in steps of 25, 2000 bootstrap replicates were sampled with replacement and analyzed using the 3-predictor logistic regression model (outcome = death). Power was calculated as the proportion of statistically significant steroid treatment effects (*P* < 0.05) in each set of 2000 replicates.^15^

Finally, our statistical analysis included a binomial test of proportions to estimate the sample size required for a prospective, randomized, controlled trial of corticosteroid treatment.

Multivariate logistic regression, bootstrap analysis, and binomial test of proportions were performed using R version 3.20.

## RESULTS

Forty-nine patients with blastomycosis and hypoxic respiratory failure received care in an ICU during the study period. Of these, 3 did not require mechanical (including noninvasive) ventilation and were excluded from further analysis. In 3 other studies, attribution of respiratory failure to blastomycosis could not be definitively established: in addition to proven blastomycosis, 1 patient each had disseminated adenocarcinoma of the lung, infective endocarditis with cardiogenic shock, and a disseminated herpes virus infection.

**FIGURE 1:**
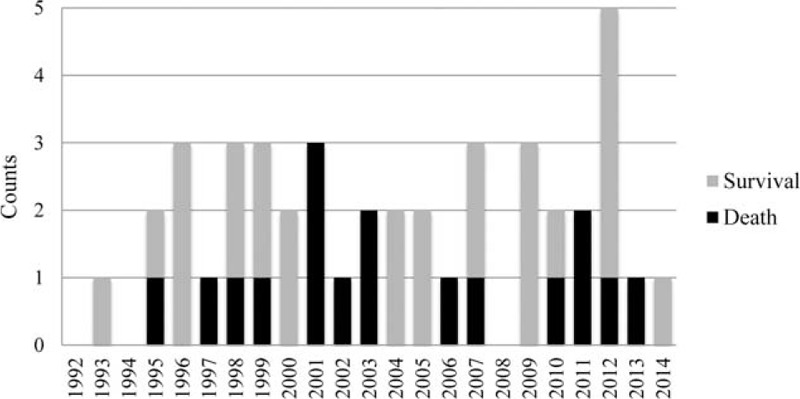
Timeline of cases of acute respiratory distress syndrome caused by blastomycosis managed in Manitoba intensive care units and their outcomes.

Forty-three patients with ARDS caused by blastomycosis were thus included in our analysis (see Table, Supplemental Content, for individual patient details). The timeline of cases and their outcomes is shown in Figure 1. Demographic and background characteristics are summarized in Table 2.

**TABLE 2:**
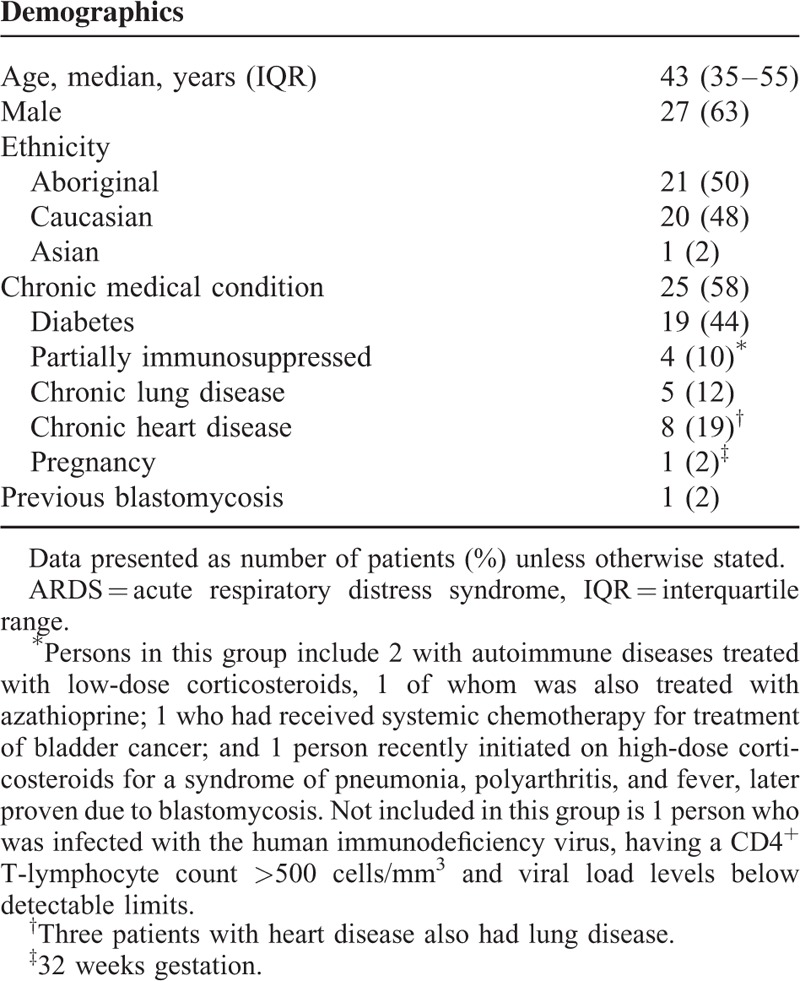
Demographic and Background Characteristics of 43 Patients With ARDS Caused by Blastomycosis

Aboriginal persons, who comprised half of our cohort, were younger than non-Aboriginal persons (median age 41 years, interquartile range [IQR] 25–49 vs 54 years, IQR 40–64; *P* = 0.04). There were, however, no differences in sex, province of residence, chronic medical conditions (including diabetes), clinical features (including presence of extrapulmonary disease and severity of ARDS or critical illness), management or outcome between ethnicities (data not shown).

Clinical characteristics of patients are presented in Table 3. In 11 patients (26%), an extrapulmonary complaint was the reason for presenting to care, with respiratory failure occurring subsequently. The median duration from initial presentation to care until respiratory failure requiring mechanical ventilation was 3 days (IQR 2–6). There was no significant difference in duration from presentation to respiratory failure according to whether the presenting complaint was pulmonary or extrapulmonary.

**TABLE 3:**
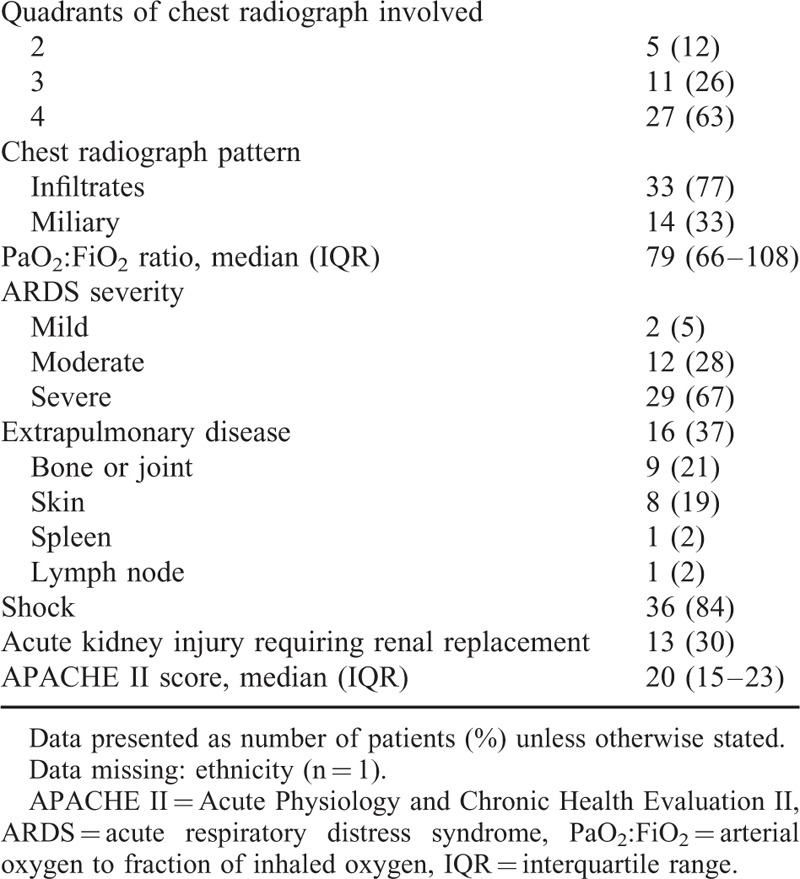
Clinical Characteristics and Diagnostic Findings of 43 Patients With ARDS Caused by Blastomycosis

Pulmonary arterial hemodynamic measurements were available for 18 patients (42%). The median pulmonary wedge pressure was 15 mm Hg (IQR 13–18). Nine patients (50%) had pulmonary wedge pressures ≥18 mm Hg, although none had clinical signs of heart failure.

Management and outcome details of patients are summarized in Table 4. Patients who received rescue therapy with corticosteroids or ECMO did not differ significantly from other patients by age, sex, ethnicity, province of residence, presence of chronic medical conditions, or critical illness severity (data not shown). Patients treated with corticosteroids were more likely to have severe ARDS (18/22 who received corticosteroids had severe ARDS, vs 11/21 who did not receive corticosteroids; *P* = 0.05). There were no differences in corticosteroid dosing, timing, or duration according to outcomes (data not shown).

**TABLE 4:**
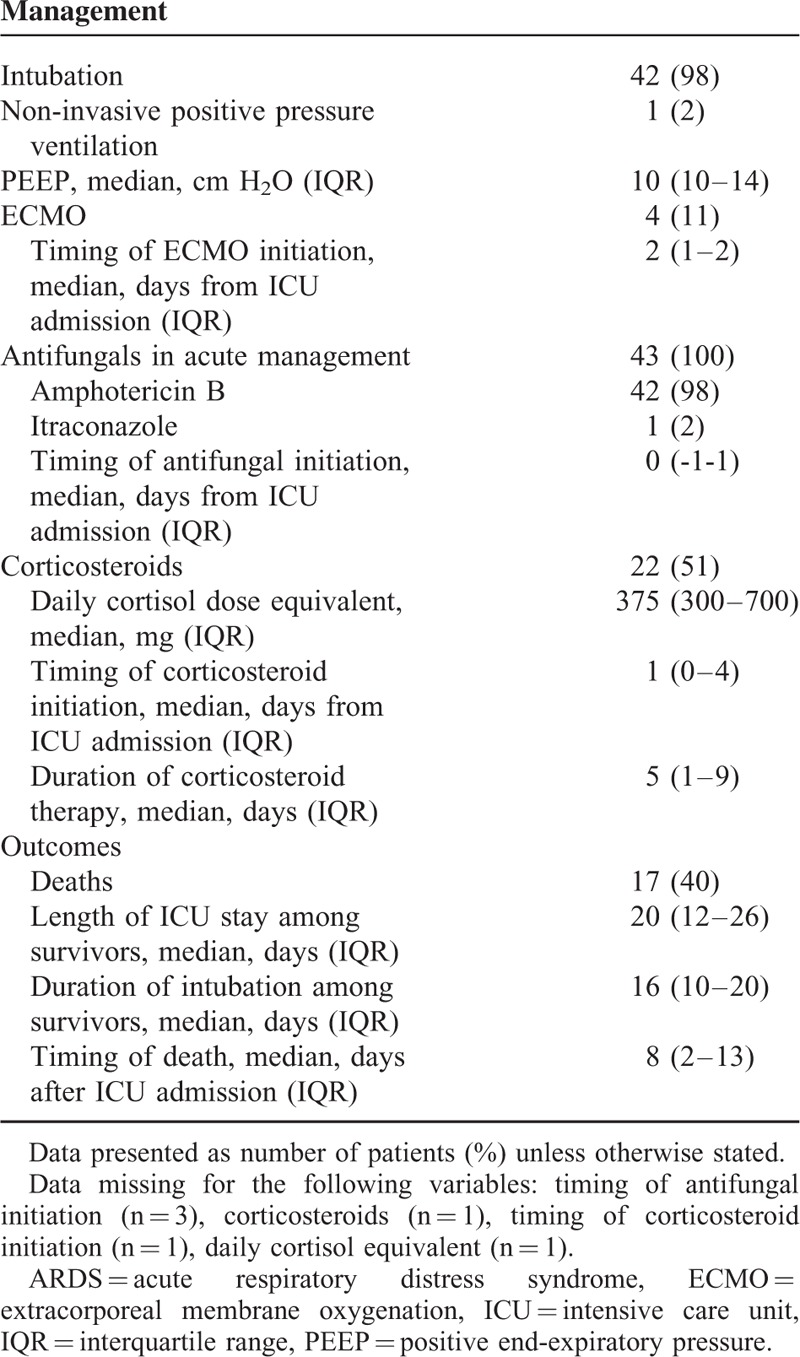
Management and Outcomes of 43 Patients With ARDS Caused by Blastomycosis

Analyses of select disease and management variables with mortality outcomes are shown in Table 5. In univariate analysis, critical illness severity and presence of cardiopulmonary disease emerged as being significantly associated with death. Multivariate logistic regression assessed the impact of corticosteroid treatment, critical illness disease severity, and underlying cardiopulmonary disease on outcome (death). Although corticosteroid therapy was not significantly associated with outcome in univariate analysis, it was included in the multivariate analysis because of an a priori selection as a variable of interest. In terms of sample size, the 3-predictor model with 5.7 events per variable should be considered exploratory.^16^ Although not shown here, the effect estimates from this model were unchanged in separate 2-predictor models (corticosteroid treatment + critical illness disease severity and corticosteroid treatment + underlying cardiopulmonary disease).

**TABLE 5:**
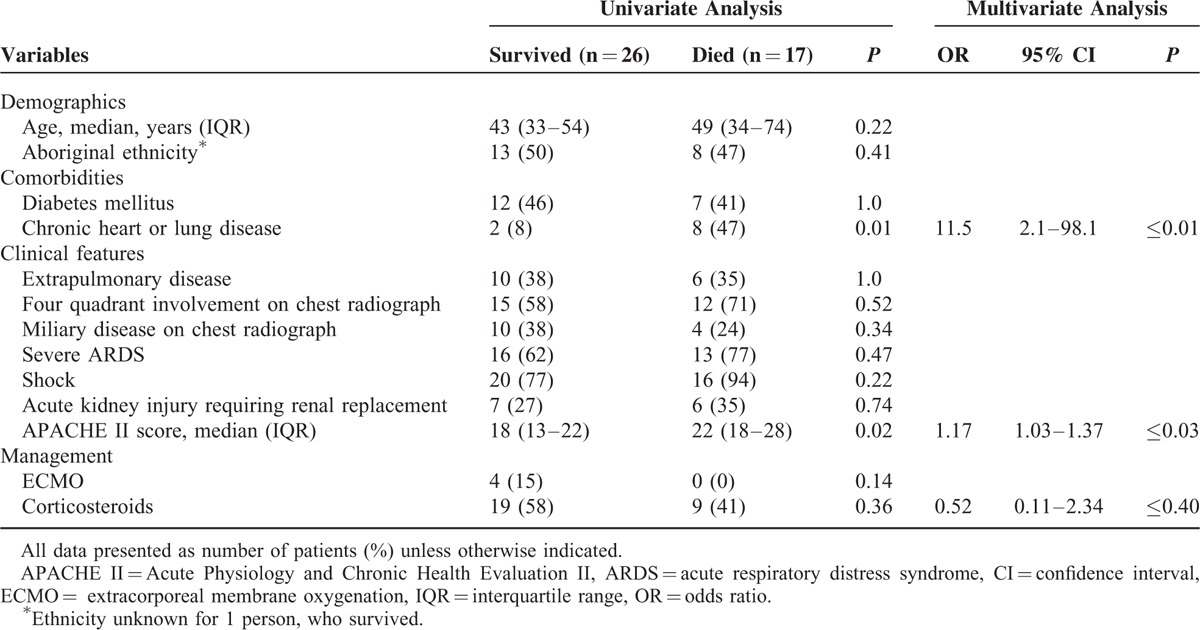
Univariate and Multivariate Analyses of Select Variables With Outcome in Patients With ARDS Caused by Blastomycosis

The observed reduction in odds of mortality with corticosteroid treatment (odds ratio [OR] 0.52, 95% confidence interval [CI] 0.11–2.34) was not statistically significant. Bootstrap resampling indicated that approximately 500 subjects would be required to confirm the observed protective effect with 80% power and a significance level of 0.05.

Assuming 48% mortality in those who did not receive corticosteroids (based on our data, in which death occurred in 10 of 21 such persons), a prospective, randomized controlled trial of corticosteroid treatment might also be considered. Based on the binomial test of proportions with a significance level of 0.05, a power of 80%, a 2-tailed alternative hypothesis, and a 1:1 randomization scheme, a total of 292 subjects would be required to demonstrate a 33% reduction in mortality, with 390 subjects needed for 90% power.

## DISCUSSION

This study summarizes the Manitoba experience in caring for critically ill patients with ARDS caused by blastomycosis over a 23-year period, from 1992 to 2014, and builds on the knowledge obtained from smaller case series from Wisconsin,^2^ Tennessee,^3^ Mississippi,^4^ and Indiana.^5^

Previous studies have suggested higher rates of blastomycosis in Aboriginal persons,^9,17–19^ and several studies have suggested increased disease severity in Aboriginal compared with non-Aboriginal persons: in Northwest Ontario, Aboriginal persons were more likely to be hospitalized with respiratory complications of blastomycosis^20^; and in the United States, native American persons were reported more likely to die of blastomycosis than white persons, with an age-adjusted mortality rate ratio of 4.13.^21^ In the present study, Aboriginal persons comprised one-half of the study population, although in 2011 (the most recent census data), Aboriginal persons comprised just 16.7% and 19.7% of the populations of the province of Manitoba and the region of Northwest Ontario, respectively.^22^ Aboriginal persons with ARDS caused by blastomycosis were younger than non-Aboriginal persons, which is consistent with another report from Northwest Ontario,^19^ and also census data on age distribution in these respective populations.^13^ Other significant differences, including outcomes, were not detected between ethnic groups.

Diabetes was common, occurring in 44% of the group, including over three-quarters of those with underlying medical conditions. Similarly, our group previously reported diabetes to be more common amongst hospitalized patients with pulmonary blastomycosis who required ICU care than those who did not (36% vs 16%; *P* = 0.02),^18^ and investigators from Indiana found diabetes to be a risk factor for ICU admission on multivariate analysis (OR 2.9, *P* = 0.04).^5^ These data suggest that persons with diabetes complicated by blastomycosis may be at increased risk of progression to respiratory failure and ARDS. In contrast, smaller case series of ARDS caused by blastomycosis have not reported high rates of diabetes,^2–4^ and large epidemiological studies have suggested that diabetes may, in fact, be protective against the development of ARDS (in processes other than blastomycosis).^23,24^ Alternatively, ARDS is a heterogeneous condition, and the pathophysiology of ARDS caused by blastomycosis may be different than ARDS from other causes.

One-third of patients had both pulmonary and extrapulmonary disease. Nearly all (93%) patients with miliary disease on chest radiograph also had extrapulmonary disease—a finding consistent with a previous study also including non-ICU patients.^25^ One interpretation of these observations is that the pathogeneses of nonmiliary pulmonary blastomycosis and miliary pulmonary blastomycosis may differ, representing endobronchial spread and hematogenous dissemination to the lungs after reactivation of extrapulmonary disease, respectively. Our group has previously reported reticulonodular (miliary) disease on chest radiograph to be a risk factor for ICU admission among patients with pulmonary blastomycosis,^18^ and it may be that acute lung injury and ARDS are more likely to follow miliary than nonmiliary disease.

Beyond the prompt institution of antifungal therapy, it is currently unknown whether optimal management of persons with ARDS caused by blastomycosis differs from that of persons with ARDS from other causes. ARDS is caused by a heterogeneous group of diseases,^26^ and it is at least possible that therapies not shown to be beneficial in trials of all persons with ARDS may be beneficial for persons with ARDS caused by blastomycosis. For instance, although prospective, randomized controlled trials of corticosteroids have failed to demonstrate a mortality benefit in the treatment of early or late phases of ARDS, optimism for a role for corticosteroids in ARDS caused by blastomycosis has been fueled by anecdotal reports,^5,8,27^ in addition to the demonstrated mortality benefit in HIV-infected patients with severe respiratory failure due to *Pneumocystis jiroveci*, another fungal pneumonia.^28^ Conversely, the role of corticosteroids in ARDS caused by tuberculosis, a mycobacterial infection also causing granulomatous disease, is uncertain.^29^

In the current study, approximately half of those with ARDS were treated with corticosteroids. A multivariate logistical regression model did not detect a significant association between outcome and corticosteroid use, even after adjustment for critical illness severity and comorbidity. While it is possible that a difference could become statistically significant with larger numbers, our bootstrap analysis suggested that the number of subjects needed for such a study (500) is impractical.

Extracorporeal membrane oxygenation is increasingly recognized as a valuable therapy in the management of ARDS,^30^ though few reports exist of ECMO in the care of patients with ARDS, specifically caused by blastomycosis.^31–33^ Four patients in this series were successfully managed with ECMO; these were recently reported in detail.^31^ Whereas these small numbers fall short of statistical significance, they do suggest that a role for ECMO in ARDS caused by blastomycosis should be explored further.^34^

The mortality rate of 40% in this series is lower than the 47% to 89% reported in previous series of ARDS caused by blastomycosis.^2–5^ Possible explanations include smaller sample sizes in previous series; differences in inclusion criteria due to changing definitions of ARDS^35^ (whereas this study used the Berlin definition to define ARDS,^10^ another^4^ used the American-European Consensus Conference definition,^36^ and 2 others^2,4^ used Murray Lung Injury Score ^37^); heightened awareness by clinicians in our geographic area, contributing to earlier diagnosis and antifungal therapy; differences in the use of adjunctive therapies; or improvements in resuscitation and intensive care. Most notable among the latter is the introduction of lung-protective ventilation—low tidal volume and high positive end-expiratory pressure to minimize barotrauma—which has been demonstrated to improve mortality in ARDS.^38^ Expectedly, survival was inversely related to critical illness severity, but, surprisingly, not to ARDS severity, number of affected quadrants on chest radiograph, or shock. One possibility is that an association between ARDS severity and mortality was attenuated by the effect of underlying cardiopulmonary disease; patients with these premorbid conditions were similarly distributed across severe and nonsevere ARDS groups, and their outcomes were worse than other patients without these conditions.

The limitations of this study are consequences of the retrospective methodology. Data on potential sources of exposure to *Blastomyces* spp. were unavailable. Because symptoms were often insidious and protean at onset, timelines of disease progression (and possible missed opportunities for early diagnosis and intervention) were unavailable. Another limitation is the absence of robust data on ventilation strategies. Multiple ventilation strategies were often employed, with changes frequently occurring on a daily basis, based upon the patient's clinical status, and consequently it was impractical to incorporate these variables in our analysis. Our definition of corticosteroid administration may have been overly restrictive, which may have overlooked the effects of lower corticosteroid doses. Additionally, data regarding possible adverse events, particularly those related to corticosteroids, were not systematically collected, and thus we cannot comment on morbidity associated with this therapy. Long-term follow-up data were also unavailable, and so this study is also unable to comment on the late morbidity and mortality from ARDS caused by blastomycosis.

Another possible limitation of this study is heterogeneity in management that is a consequence of the long study period, since diagnostic and/or management practices may have changed over time. Critical care has advanced over the 23 years of this study, and it is plausible that these changes—incremental as they may be—could confound our findings. Conversely, specific advances in the care of patients with blastomycosis have been modest.^6,39^ Diagnosis remains predicated on cytopathological examination with fungal stains and culture of clinical specimens, whereas ancillary diagnostics, like serology or antigen tests, have yet to be widely adopted and were not employed at our center. Therapeutic advances for severe blastomycosis have also been modest, with amphotericin B deoxycholate replaced by less nephrotoxic lipid formulations, and itraconazole replacing fluconazole and ketoconazole as the triazole of choice for consolidation phases of therapy.

Finally, it should be noted that this study reflects the experience at referral hospitals serving a restricted geographic region (Manitoba and Northwest Ontario), and both patient and pathogen characteristics may differ from other settings. Aboriginal persons comprised half of the patients in our series, and, as discussed above, it is possible that risks of complications due to blastomycosis may be different than for other ethnic groups. Moreover, specific local challenges for the referral population include limited access to healthcare services for persons living in remote communities^40^—a problem compounded for Aboriginal persons living on reserves.^41^ This may be of relevance if early diagnosis and therapy for blastomycosis can avert or attenuate the complication of ARDS. In addition, recent studies have highlighted genetic variation of *Blastomyces* spp.^42,43^ In our study, the phenotypic and, later, genotypic methods used for organism identification were unable to distinguish between *Blastomyces dermatitidis* and *Blastomyces gilchristii,* a recently described cryptic species that predominates in Northwest Ontario, among other areas.^43^ Clinical differences between these species have not been clearly established. Although a recent case report from Northwest Ontario highlighted the potential of *B gilchristii* to cause ARDS,^44^ it is currently unknown if differences exist in pathogenesis, virulence, or response to therapy.

Even though this study, representing 23 years of experience providing care for a region of hyperendemicity, is the largest reported series of persons with ARDS caused by blastomycosis, there were insufficient subjects to allow for conclusions to be drawn about the role of adjuvant corticosteroids in the management of this disease. However, given the low incidence of blastomycosis, the infrequency of the complication of ARDS, and the large cohort of patients needed to detect statistical significance, either in a prospective trial or retrospective study, a more definitive estimate of treatment effect is likely impractical. Thus, management of patients with ARDS caused by blastomycosis, including the use of rescue therapies such as adjunctive corticosteroids and/or ECMO, should be guided by clinical judgment on a case-by-case basis.

## Supplementary Material

Supplemental Digital Content
