# Supplementary material for: Management and Outcomes of Acute Respiratory Distress Syndrome Caused by Blastomycosis: A Retrospective Case Series
Source: Medicine (Baltimore). 2016 May 6;95(18):e3538. doi: 10.1097/MD.0000000000003538 (PMC4863776; doi:10.1097/MD.0000000000003538)
Supplement: Supplemental Digital Content [file medi-95-e3538-s001.docx]

Supplementary Table 1: Demographics, clinical characteristics, management, and outcomes of patients with acute respiratory distress syndrome caused by blastomycosis

| **Case** | **Year** | **Age** | **Sex** | **Ethnicity** | **Comorbidities** | **Site of Infection** | **Chest X-ray** | | **Invasive Ventilation** | **PaO_2_:FiO_2_** | **PEEP** | **ARDS Severity** | **Shock** | **Dialyzed** | **ECMO** | **Corticosteroids** | | | | **Antifungals** | | **Outcome** | **Timing**^b^ | |
| --- | --- | --- | --- | --- | --- | --- | --- | --- | --- | --- | --- | --- | --- | --- | --- | --- | --- | --- | --- | --- | --- | --- | --- | --- |
|  |  |  |  |  |  |  | **Quad-rants** | **Lesion** |  |  |  |  |  |  |  | **Yes/ No** | **Dose^a^ (mg)** | **Timing**^b^ | **Duration (days)** | **AMB** | **Timing**^b^ |  | **Death** | **ICU Discharge** |
| 1 | 1993 | 28 | M | Aboriginal | Nil | Lung, Knee^c^ | 4 | Miliary | ✓ | 80 | 10 | Severe | ✕ | ✕ | ✕ | ✓ | 200 | 4 | 6 | ✓ | -1 | Survived |  | 10 |
| 2 | 1995 | 43 | F | Caucasian | Nil | Lung | 4 | Infiltrate | ✓ | 79 | 10 | Severe | ✓ | ✕ | ✕ | ✕ |  |  |  | ✓ | 3 | Died | 4 |  |
| 3 | 1995 | 18 | M | Aboriginal | Nil | Lung, Foot^c^, Soft Tissue | 4 | Miliary | ✓ | 87 | 7.5 | Severe | ✓ | ✕ | ✕ | ✓ | 1000 | 2 | 4 | ✓ | 1 | Survived |  | 13 |
| 4 | 1996 | 54 | M | Caucasian | DM | Lung | 3 | Infiltrate | ✓ | 70 | 10 | Severe | ✓ | ✕ | ✕ | ✓ | 150 | 5 |  | ✓ | -4 | Survived |  | 16 |
| 5 | 1996 | 64 | F | Caucasian | Nil | Lung | 2 | Infiltrate | ✓ | 75 | 7.5 | Severe | ✕ | ✕ | ✕ | ✕ |  |  |  | ✓ | 0 | Survived |  | 24 |
| 6 | 1996 | 42 | M | Caucasian | Nil | Lung, Knee^c^ | 4 | Miliary | ✓ | 101 | 12.5 | Mod. | ✓ | ✕ | ✕ | ✕ |  | 14 |  | ✓ | 0 | Survived |  | 26 |
| 7 | 1997 | 44 | M | Aboriginal | Nil | Lung, Foot^c^, Skin | 4 | Miliary | ✓ | 70 | 12.5 | Severe | ✓ | ✓ | ✕ | ✓ | 1600 | 8 | 6 | ✓ | 1 | Died | 15 |  |
| 8 | 1998 | 62 | F | Aboriginal | DM, heart disease, cirrhosis, schizophrenia | Lung, Lymph nodes | 4 | Infiltrate | ✓ | 43 | 7.5 | Severe | ✓ | ✕ | ✕ | ✕ |  |  |  | ✕ | 1 | Died | 2 |  |
| 9 | 1998 | 55 | M | Caucasian | Heart and kidney disease, bladder cancer | Lung, Rib, Retrosternal abscess | 4 | Miliary | ✓ | 100 | 7.5 | Severe | ✓ | ✕ | ✕ | ✓ | 375 | 1 | 12 | ✓ | -2 | Survived |  | 36 |
| 10 | 1998 | 63 | M | Caucasian | DM | Lung | 2 | Infiltrate | ✓ | 122 | 7.5 | Mod. | ✕ | ✕ | ✕ | ✕ |  |  |  | ✓ | -1 | Survived |  | 16 |
| 11 | 1999 | 24 | F | Aboriginal | DM | Lung | 3 | Infiltrate | ✓ | 110 | 15 | Mod. | ✕ | ✕ | ✕ | ✓ | 300 | 0 | 6 | ✓ | -2 | Survived |  | 9 |
| 12 | 1999 | 55 | F | Caucasian | Nil | Lung | 3 | Infiltrate | ✓ | 105 | 5 | Mod. | ✓ | ✕ | ✕ | ✕ |  |  |  | ✓ | -3 | Survived |  | 16 |
| 13 | 1999 | 54 | M | Caucasian | DM, heart & cerebrovascular disease | Lung | 3 | Infiltrate | ✓ | 66 | 15 | Severe | ✓ | ✕ | ✕ | ✕ |  |  |  | ✓ | 0 | Died | 8 |  |
| 14 | 2000 | 54 | F | Aboriginal | DM, cerebrovascular disease | Lung | 2 | Infiltrate | ✕ | 153 | 7.5 | Mild | ✕ | ✕ | ✕ | ✕ |  |  |  | ✓ | 0 | Survived |  | 5 |
| 15 | 2000 | 39 | F | Caucasian | DM | Lung | 4 | Infiltrate | ✓ | 38 | 12.5 | Severe | ✓ | ✕ | ✕ | ✓ | 5000 | 2 | 16 | ✓ | 9 | Survived |  | 22 |
| 16 | 2001 | 82 | M | Aboriginal | Heart disease | Lung | 2 | Infiltrate | ✓ | 56 | 8 | Severe | ✓ | ✕ | ✕ | ✕ |  |  |  | ✓ | -1 | Died | 10 |  |
| 17 | 2001 | 77 | F | Caucasian | Cerebrovascular disease | Lung | 4 | Infiltrate | ✓ | 108 | 10 | Mod. | ✓ | ✕ | ✕ | ✕ |  |  |  | ✓ | 5 | Died | 13 |  |
| 18 | 2001 | 20 | M | Asian | Nil | Lung, Ankle^c^ | 4 | Miliary | ✓ | 64 | 19 | Severe | ✓ | ✕ | ✕ | ✓ | 300 | 0 | 10 | ✓ | 3 | Died | 9 |  |
| 19 | 2002 | 20 | M | Aboriginal | Nil | Lung | 4 | Infiltrate | ✓^d^ | 60 | 10 | Severe | ✓ | ✕ | ✕ | ✕ |  |  |  | ✓ | -3 | Died | 5 |  |
| 20 | 2003 | 49 | M | Aboriginal | DM, heart and lung disease | Lung, Skin | 4 | Infiltrate | ✓ | 42 | 10 | Severe | ✓ | ✕ | ✕ | ✕ |  |  |  | ✓ | 1 | Died | 2 |  |
| 21 | 2003 | 49 | M | Aboriginal | Alcohol abuse | Lung | 2 | Infiltrate | ✓^d^ | 44 | 15 | Severe | ✓ | ✓ | ✕ | ✓ | 150 | 1 | 1 | ✓ | -1 | Died | 1 |  |
| 22 | 2004 | 48 | M | Caucasian | DM | Lung, Skin, Spleen^c^ | 4 | Infiltrate & Miliary | ✓ | 121 | 14 | Mod. | ✓ | ✓ | ✕ | ✕ |  |  |  | ✓ | -1 | Survived |  | 67 |
| 23 | 2004 | 45 | M | Aboriginal | DM, alcohol abuse, liver disease, chronic pancreatitis | Lung | 3 | Infiltrate | ✓ | 71 | 14 | Severe | ✓ | ✕ | ✕ | ✕ |  |  |  | ✓ | 7 | Survived |  | 83 |
| 24 | 2005 | 43 | M | Aboriginal | DM | Lung | 4 | Infiltrate | ✓ | 106 | 10 | Mod. | ✕ | ✓ | ✕ | ✕ |  |  |  | ✓ | 31 | Survived |  | ? |
| 25 | 2005 | 39 | F | Caucasian | Nil | Lung, Skin | 4 | Infiltrate & Miliary | ✓ | 154 | 10 | Mod. | ✓ | ✕ | ✕ | ✕ |  |  |  | ✓ | 1 | Survived |  | 9 |
| 26 | 2006 | 40 | F | Aboriginal | DM, heart and kidney disease | Lung | 3 | Infiltrate | ✓ | 87 | 15 | Severe | ✓ | ✓ | ✕ | ✓ | 1000 | 5 | 7 | ✓ | 1 | Died | 12 |  |
| 27 | 2007 | 32 | F | Aboriginal | DM, pregnancy^e^ | Lung | 4 | Miliary | ✓ | 70 | 17.5 | Severe | ✓ | ✕ | ✕ | ✓ | 200 | 1 | 1 | ✓ | 0 | Died | 1 |  |
| 28 | 2007 | 45 | F | Aboriginal | DM, kidney disease | Lung, Knee^c^ | 4 | Miliary | ✓ | 84 | 14 | Severe | ✓ | ✓ | ✕ | ✓ | 375 | 4 | 5 | ✓ | 1 | Survived |  | 20 |
| 29 | 2007 | 26 | M | Aboriginal | Nil | Lung | 3 | Infiltrate | ✓ | 76 | 10 | Severe | ✓ | ✕ | ✕ | ✓ | 800 | 0 | 7 | ✓ | -1 | Survived |  | 20 |
| 30 | 2009 | 55 | F | Aboriginal | DM, heart and lung disease, rheumatoid arthritis^f,g^ | Lung | 4 | Infiltrate | ✓ | 108 | 12.5 | Mod. | ✓ | ✕ | ✕ | ✓ | 400 | 0 | 5 | ✓ | 4 | Survived |  | 26 |
| 31 | 2009 | 41 | M | Aboriginal | Nil | Lung, Vertebrae^c^, Paraspinal muscles | 4 | Miliary | ✓ | 148 | 10 | Mod. | ✓ | ✕ | ✕ | ✕ |  |  |  | ✓ | 1 | Survived |  | 18 |
| 32 | 2009 | 22 | F | Aboriginal | Nil | Lung | 4 | Infiltrate | ✓ | 70 | 16 | Severe | ✓ | ✓ | ✓ | ✓ | 300 | 1 | 20 | ✓ | 1 | Survived |  | 22 |
| 33 | 2010 | 58 | F | Caucasian | Nil | Lung, Skin, Elbow^c^, Soft tissue | 4 | Infiltrate & Miliary | ✓ | 80 | 14 | Severe | ✓ | ✕ | ✕ | ✓ | 600 | 3 | 1 | ✓ | 0 | Survived |  | 10 |
| 34 | 2010 | 36 | M | Caucasian | HIV^h^, pulmonary hypertension, chronic thromboembolic disease | Lung | 3 | Infiltrate | ✓^d^ | 77 | 10 | Severe | ✓ | ✕ | ✕ | ✕ |  |  |  | ✓ | 0 | Died | 3 |  |
| 35 | 2011 | 31 | M | Caucasian | DM | Lung, Skin | 4 | Infiltrate | ✓ | 63 | 16 | Severe | ✓ | ✓ | ✕ | ✕ |  |  |  | ✓ | 0 | Died | 15 |  |
| 36 | 2011 | 84 | M | Caucasian | Nil | Lung, Skin, Knee^c^ | 4 | Miliary | ✓ | 170 | 14 | Mod. | ✓ | ✓ | ✕ | ✓ | unknown | 1 | 1 | ✓ | 1 | Died | 2 |  |
| 37 | 2012 | 43 | M | Caucasian | DM, cerebrovascular disease | Lung, Knee^c^ | 4 | Infiltrate & Miliary | ✓ | 123 | 10 | Mod. | ✓ | ✕ | ✕ | ✕ |  |  |  | ✓ | 0 | Survived |  | 9 |
| 38 | 2012 | 35 | M | Unknown | Nil | Lung | 3 | Infiltrate | ✓ | 63 | 10 | Severe | ✓ | ✕ | ✕ | ✕ | 300 | 0 | 1 | ✓ | -2 | Survived |  | 27 |
| 39 | 2012 | 39 | M | Aboriginal | Nil | Lung | 4 | Infiltrate | ✓ | 61 | 22 | Severe | ✓ | ✓ | ✓ | ✓ | 462 | 0 | 3 | ✓ | 0 | Survived |  | 21 |
| 40 | 2012 | 78 | M | Caucasian | Heart & lung disease | Lung | 4 | Infiltrate | ✓ | 111 | 8 | Mod. | ✓ | ✕ | ✕ | ✕ |  |  |  | ✓ | 21 | Died | 48 |  |
| 41 | 2012 | 21 | F | Aboriginal | Nil | Lung | 3 | Infiltrate | ✓ | 78 | 12 | Severe | ✓ | ✓ | ✓ | ✓ | 300 | 0 | 1 | ✓ | 2 | Survived |  | 49 |
| 42 | 2013 | 71 | M | Caucasian | DM, lung disease, polymyalgia rheumatica^f^ | Lung | 4 | Infiltrate | ✓ | 212 | 10 | Mild | ✕ | ✕ | ✕ | ✓ | 600 | 0 | 1 | ✓ | -1 | Died | 13 |  |
| 43 | 2014 | 39 | M | Caucasian | DM, hemochromatosis, adrenal insufficiency^f^, stiff person's syndrome | Lung | 3 | Infiltrate | ✓ | 70 | 14 | Severe | ✓ | ✓ | ✓ | ✓ | 600 | 1 | 16 | ✓ | 0 | Survived |  | 25 |

Notes:

^a^daily cortisol dose equivalent (mg); ^b^timing in days from ICU admission; ^c^denotes chief complaint at presentation (in cases where pulmonary and extra-pulmonary disease present); ^d^independent lung ventilation attempted;

^e^32 weeks pregnant; ^f^treated with low dose corticosteroids;  ^g^treated with azathioprine; ^h^CD4>350 cells/mm^2^ and viral load undetectable;

Abbreviations: AMB: amphotericin B; DM: Diabetes mellitus; ECMO: extracorporeal membrane oxygenation; F: female; ICU: intensive care unit; itra.: itraconazole; M: male; Mod: moderate; PaO2:FiO2: Partial pressure of arterial oxygen to inhaled oxygen concentration ratio; PEEP: positive end expiratory pressure
